# Nurses Practice of Hand Hygiene in Hiwot Fana Specialized University Hospital, Harari Regional State, Eastern Ethiopia: Observational Study

**DOI:** 10.1155/2018/2654947

**Published:** 2018-04-16

**Authors:** Nefsu Awoke, Biftu Geda, Aseb Arba, Tiwabwork Tekalign, Kebreab Paulos

**Affiliations:** ^1^Department of Nursing, College of Health Science and Medicine, Wolaita Sodo University, Wolaita Sodo, Ethiopia; ^2^School of Nursing and Midwifery, College of Health and Medical Sciences, Haramaya University, Harar, Ethiopia; ^3^Department of Midwifery, College of Health Science and Medicine, Wolaita Sodo University, Wolaita Sodo, Ethiopia

## Abstract

**Background:**

Nurses, who are the majority, can contaminate their hands with different types of microorganism during “clean” activities (e.g., lifting a patient; taking a patient's pulse, blood pressure, or oral temperature; or touching a patient's hand, shoulder, or groin). Yet good hand hygiene, the simple task of cleaning hands at the right time and in the right way, can reduce HCAIs that are transmitted by healthcare workers' hands.

**Method:**

Observational study conducted among nurses by observational tool which was adopted from WHO observational tool. And finally compliance was calculated as a percentage (i.e., compliance% = (observed hand hygiene action (HHA) ÷ hand hygiene opportunity (O)) × 100). The data were first coded, entered, and cleaned using EpiData statistical software version 3.1 and then exported into SPSS statistical software version 22 for analysis. Data were presented using descriptive statistics.

**Result:**

A total of 110 study participants were observed who gave a response rate of 94.8%. Total of 3902 opportunities and 732 hand hygiene actions were observed with overall compliance of 18.7%. The highest 22.9% hand hygiene practice was observed “before clean∖aseptic procedure.” Highest 19.6% compliance was recorded at night shift and 22.7% in ICU ward of the hospital. Alcohol based hand rub was a major means of method used to clean hands.

**Conclusion and Recommendation:**

Observed practice of hand hygiene was poor. Lack of training, conveniently located sink, hand washing agents, and lack of time were major reasons for not practicing hand hygiene. Successful promotion of hand hygiene through instituting system change (e.g., making hand hygiene products available at the point of care) should be considered.

## 1. Background

Hand hygiene refers to removal of microorganisms which are transient or killing them and avoiding of visible dirty from hands without causing any harm to skin by using different techniques and hand washing agents [1]. Transient flora found in superficial skin were acquired during contact within healthcare environment such as between contaminated equipment, patient, and health professionals. These types of flora were more easily to be removed by simple practice of hand hygiene but resident types of floras found in deeper part of skin were difficult to remove through hand hygiene [1, 2].

Hands of healthcare workers act as vehicle for the transmission of healthcare-associated pathogens [3] by continuously touching different substances and surfaces such as waste, body fluids, mucous membranes, food, their own body, and patients skin which can be intact or nonintact, different intimate objects while performing healthcare activities [4] at this time their hands were colonized by different groups of pathogens that are drug-resistant such as* Clostridium difficile*, gram-negative bacteria,* S. aureus*,* Enterococcus*, and* Candida* spp. [2, 4, 5].

Healthcare-associated infection (HCAI) places a serious disease burden and has a significant economic impact on patients and healthcare systems throughout the world [6]. A number of pathogens were found to be as a major cause of hospital-acquired infection such as* Klebsiella* spp.,* S. aureus*,* Pseudomonas aeruginosa*,* E. coli*,* Enterobacter* spp.,* Streptococcus pneumonia*,* Proteus* spp.,* Citrobacter* spp.,* Klebsiella pneumonia*,* Acinetobacter* spp., and* Serratia* spp. [7]. Even though the practice of hand hygiene in most occasions was low still it was considered as a single most effective method to tackle the new emerging burden posed by drug resistance microorganisms which were challenges in healthcare institutions by causing many suffering [3].

It is estimated that approximately 30% of healthcare providers report symptoms or signs of dermatitis involving their hands, and as many as 85% give a history of having skin problems after performing healthcare activities [1]. Yet good hand hygiene, the simple task of cleaning hands at the right time and in the right way, can reduce HCAIs that are transmitted by healthcare workers' hands which become progressively colonized by germs and potential pathogens during patient care [4].

Different studies identified different factors that hinder practice of hand hygiene effectively such as using glove, unavailability and inaccessibility of alcohol-based hand rub, inadequate water supply, absence of detergent/soap, unavailability and inaccessibility of wash basins/sinks, lack of clean towels, poor quality of soap, and lack of hand lotion/lubricants [8–10].

Nurses, who are the majority, can contaminate their hands with 100–1,000 colony-forming units (CFUs) of* Klebsiella *spp. during “clean” activities (e.g., lifting a patient; taking a patient's pulse, blood pressure, or oral temperature; or touching a patient's hand, shoulder, or groin) [5]. Therefore, this study aimed to assess nurses practice of hand hygiene and identify factors that hinder hand hygiene practice in Hiwot Fana Specialized University Hospital, Harari Regional State, Eastern Ethiopia.

## 2. Methods

### 2.1. Study Area and Study Design

Hospital based observational cross-sectional study was conducted from July 3 to July 28, 2017, at the inpatient department of HFSUH in Harari Region, Harar town, Eastern Ethiopia, from July 3 to July 28, 2017. Harari Regional State is located 515 km away from A.A with estimated area of 334 square kilometers.

### 2.2. Study Population and Sample Size

The observation was conducted on all nurses (116) who were working in inpatient department (Medical, Surgical, Pediatrics, Maternity, and ICU) of HFSUH.

### 2.3. Sampling Procedure

Consecutive sampling technique was applied to obtain hand hygiene opportunities.

### 2.4. Data Collection Instruments and Procedure

Observational checklist and self-administered questionnaires were tools used for data collection. Observational checklist which was adapted from WHO observational tool was used to assess hand hygiene practice [11]. The checklist was pretested before actual field work. The observational checklist contains sociodemographic characteristics and hand hygiene practice based on WHO 5 indications for hand hygiene. The tool is based on the principle that when nurses do any nursing procedure and care, the nurse has a “hand hygiene opportunity” (O). When a nurse responds to this opportunity by either washing by soap and water (HW) or hand rub by alcohol (HR) a nurse has hand hygiene action (HHA), if not he/she missed action (M). An observer records opportunities observed (O) and hand hygiene action (HA); finally compliance was calculated as a percentage, (i.e., compliance% = (observed hand hygiene action (HHA) ÷ hand hygiene opportunity (O)) × 100).

Instructions on how to conduct observation were provided to data collectors and supervisors in a written protocol and in form of training before actual field work. Data were collected for 3 weeks in working hours of three shifts (morning, afternoon, and night shift) each day except weekends. The data collectors obtained informed voluntary verbal and signed consent from each respondent prior to data collection. In order to minimize the Hawthorne effect, observers did not provide details of the study procedures for nurses. The observer found a place where he/she could watch both the contacts and patient care activities and hand hygiene. Finally self-administered questionnaire was administered to identify factors that hinder hand hygiene practice of the nurses.

### 2.5. Data Processing and Analysis

The data were first coded, entered, and cleaned using EpiData statistical software version 3.1 and then exported into SPSS statistical software version 22 for analysis.

Descriptive statistics (frequency, percent, mean, standard deviation, and tables) were used to present result on hand hygiene practice and factors that hinder the practice of hand hygiene.

### 2.6. Ethical Consideration

The study protocol was approved by the Haramaya University, College of Health and Medical Sciences, Institutional Health Research Ethics Review Committee. Official letters of cooperation was written to Hiwot Fana Specialized University Hospital and concerned bodies to obtain their cooperation in facilitating the study. Information on the study was explained to the participants, including the procedures, potential risks, and benefits of the study. Informed voluntary written and signed consent were obtained from all respondents prior to the study.

## 3. Result

### 3.1. Sociodemographic Characteristics

A total of 110 study participants were observed that gave a response rate of 94.8%. The mean age (±SD) of respondents was 31.2 ± 7.5 years. Female participants account 208 (56.4%). Majority of the respondents have greater than four years of experience with 6.33 mean working years. Seventy-eight (21.1%) of the respondents were from medical ward of the hospitals (Table 1).

### 3.2. Observational Result of Hand Hygiene Practice

A total of 3902 opportunities and 732 hand hygiene actions were observed. The overall compliance was 18.7%. The compliance based on indication for hand hygiene indicated that the highest 22.9% hand hygiene practice was observed “before clean∖aseptic procedure.” Regarding the compliance in the working shift the highest 19.6% compliance was recorded at night shift and the observational data specific to the inpatient department indicates that the highest compliance 22.7% was observed in ICU ward of the hospital. Regarding opportunities observed, the highest numbers were recorded before touching patient (981), at night shift (1427), and emergency ward (781) compared to their counterparts. For each opportunity the hand hygiene action was performed by either washing with soap and water (HW) or hand rub by alcohol (HR); the highest action (179) was observed after touching a patient, in night working shift the highest (280) hand hygiene action was observed, and ICU had the highest (163) hand hygiene action compared to other wards (Table 2).

**Figure 1:**
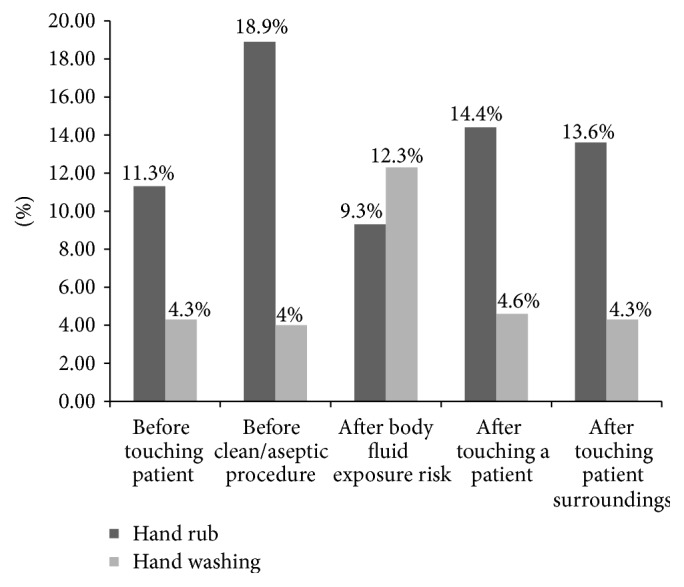
Method of hand hygiene of nurses in governmental hospitals of Harari Regional State, Eastern Ethiopia, 2017.

Concerning the method used for hand hygiene action, the majority, 111 (11.3%) among 153 actions “before touching patient,” 109 (18.9%) among 132 actions “before clean/aseptic procedure,” 136 (14.4%) among 179 actions “after touching a patient,” and 128 (13.6%) among 168 actions “after touching patient surroundings,” were performed by hand rubbing with alcohol based hand rub. Hand washing with soap and water was performed highly, 57 (12.3%) among 100 actions “after body fluid exposure” compared to any other indications of hand hygiene (Figure 1).

Reason for not practicing hand hygiene [HH] was shown by self-administered questionnaire after observation for each nurse with response rate of 100%; the majority (93 (84.5%)) of the respondents' reason was lack of training, 84 (76.4) respondents' reason was lack of conveniently located sink, and 87 (79.1) said lack of time to perform hand hygiene (Table 3).

## 4. Discussion

The results of this study in observed hand hygiene practice of nurses based on WHO indication were as follows: before touching a patient, 15.6%; before clean/aseptic procedure, 22.9%; after body fluid exposure risk, 21.6%; after touching a patient, 19%; and after touching patient surroundings, 17.9%. The overall observed hand hygiene practice was 18.7%.

Also observed practice was low compared to the finding from different studies that ranged between 21.48 and 53% in Northern India, Kuwait, South Florida, Istanbul, Saudi Arabia, and Nigeria [8, 9, 12–15]. This might be explained due to difference in the level of knowledge of nurses, wearing gloves, high workload, and institutional conditions like lack of hand hygiene resources in this study area. But it was comparable to study conducted in Gonder, 16.5% [16], but higher than study done in Addis Ababa, 3.5% (144 opportunities) [17], which might be due to higher number of opportunities being observed in this study.

Different studies indicated that hand hygiene practice among nurses before touching a patient was 41.7%, 38.6%, 43.8%, and 10% in study conducted in South Florida, Taiwan, Istanbul, and Northern India, respectively [12–14, 18].

Practice after touching patient was 74.6%, 72.1%, 42.0%, and 16% in studies conducted in Istanbul, South Florida, and Taiwan, respectively [12–14, 18].

Practice after exposure to body fluids among nurses was too 5.5%, 16.8%, and 43.5% in study conducted in Taiwan, Istanbul, and Northern India, respectively [12, 14, 18].

Practice before aseptic and clean procedure among nurses showed 6.8%, 7%, and 23.8% and hygiene practice after touching patient surroundings was 16.1%, 42.86%, and 60.1% in study conducted in Taiwan, Istanbul, and Northern India [12, 14, 18].

Generally when compared to this study the observed practice of hand hygiene was lower than this study; the difference might be due to lack of knowledge, training, and awareness about the WHO indications on hand hygiene. Even though there is socioeconomic difference between these countries the measurement tool we used was similar.

Most of the hand hygiene opportunities 781 in this study occurred in the emergency wards reflecting a high demand for hand hygiene. The highest compliance was observed in ICU, 22.7%, and the lowest in the emergency wards, 15.5%; in other wards the result indicates 16.5% in medical wards, 17.9% in surgical wards, 20.8% in pediatrics, and 20.4% in maternity.

There was a significant difference of hand hygiene practice in the observed compliance between different ward categories (*p* < 0.001), 14.7% in emergency, 40% as surgery, 43.4% at pediatrics, 45.2% at ICU/CCU, and 55% in medical wards in study conducted in Kuwait in 2009 [8]. But observational study conducted in Addis Ababa in 2012 indicated nurses working in Emergency Department had 4.9 better hand hygiene adherences compared to nurses working in surgical ward (AOR = 4.9, 95% CI 2.8–8.6, *p* < 0.001) [17]; the result of this study was low compared to research done in other countries; the possible reason might be the wards in other countries may be equipped with wand washing equipment and there may be regular availability of water and hand washing agents which are deficient in this study area.

This study was consistent with the study conducted in Southern Nigeria in which nurses who believed that facilities for hand washing were inadequate were less likely to have good handwashing practice [10]. Also study in Gonder indicated availability of sink in working ward increased the practice of hand hygiene. These might be due to the fact that presence of conveniently located sink at point of care will ease the practice of hand hygiene.

Generally, the study tried to assess the magnitude of hand hygiene practice of nurses and it can be an input for infection prevention program together with other pocket studies from different corners of the country (Ethiopia). But the study might have faced Hawthorne effect; hence observation will be used as data collection tool; the observer may have potential influence on behaviors of nurses (since this method implies that the nurses are aware of being observed) and the impact of the observer's interpretation of the definitions and the actual situation on the reliability of the data.

## 5. Conclusion and Recommendation

Observed practice of hand hygiene among nurses in Hiwot Fana specialized University Hospital was too poor. High practice was observed in ICU ward and low practice was observed in emergency ward even though the most of the hand hygiene opportunities occurred in the emergency wards reflecting a high demand for hand hygiene. Lack of training, conveniently located sink, hand washing agents, and lack of time were major reasons for not practicing hand hygiene. Successful promotion of hand hygiene through multiple strategies, which includes senior and middle management support and commitment to make hand hygiene an organizational priority and instituting system change (e.g., making hand hygiene products available at the point of care), should be considered.

## Figures and Tables

**Table 1:** Sociodemographic characteristic of nurses in Hiwot Fana Specialized University Hospital [HFSUH], Harar, Eastern Ethiopia, 2017.

Variables	Frequency	Percentage
Age		
18–25	35	31.8
26–35	63	57.3
≥36	12	10.9
Sex		
Male	49	44.5
Female	61	55.5
Educational level		
Diploma	19	17.3
BSc and above	91	82.7
Year of experience		
≤1	26	23.6
2–4	50	45.5
>4	34	30.9
Ward currently working		
Medical	31	28.2
Surgical	22	20
Pediatrics	32	29.1
Maternity	15	13.6
ICU	10	9.1

**Table 2:** Observational hand hygiene practice of Nurses in Hiwot Fana Specialized University Hospital [HFSUH], Harari, Eastern Ethiopia, 2017.

	Opportunities	HH action	Compliance (%)
Indication			
Before touching a patient	981	153	15.6
Before clean/aseptic procedure	576	132	22.9
After body fluid exposure risk	463	100	21.6
After touching a patient	943	179	19
After touching patient surroundings	939	168	17.9
*Over all*	*3902*	*732*	*18.7*
Shift			
Morning	1354	253	18.7
Afternoon	1121	199	17.8
Night	1427	280	19.6
Ward			
Medical	638	105	16.5
Surgical	750	134	17.9
Pediatrics	505	105	20.8
Maternity	511	104	20.4
ICU	717	163	22.7
Emergency	781	121	15.5

**Table 3:** Respondents' reasons for not practicing hand hygiene in Hiwot Fana Specialized University Hospital [HFSUH], Harari, Eastern Ethiopia, 2017.

Reasons for not practicing hand hygiene	Frequency	Percentage
Lack of training		
Yes	93	84.5
No	17	15.5
Fear of irritation and dryness to hand washing agents		
Yes	54	49.1
No	56	50.9
Lack of conveniently located sink		
Yes	84	76.4
No	26	23.6
Lack of hand washing agents		
Yes	79	71.8
no	31	28.2
Wearing glove		
Yes	67	60.9
No	43	39.1
Lack of time		
Yes	87	79.1
No	23	20.9

## Data Availability

The datasets used and/or analysed during the current study are available from the corresponding author on reasonable request.

## Abbreviations

ABHR: Alcohol based hand rub
HCAIs: Healthcare-associated infections
HCWs: Healthcare workers
HFSUH: Hiwot Fana Specialized University Hospital
HH: Hand hygiene
HHA: Hand hygiene action
HW: Hand washing
ICU: Intensive Care Unit.
